# Fibrogenic Gene Expression in Hepatic Stellate Cells Induced by HCV and HIV Replication in a Three Cell Co-Culture Model System

**DOI:** 10.1038/s41598-018-37071-y

**Published:** 2019-01-24

**Authors:** Abdellah Akil, Mark Endsley, Saravanabalaji Shanmugam, Omar Saldarriaga, Anoma Somasunderam, Heidi Spratt, Heather L. Stevenson, Netanya S. Utay, Monique Ferguson, MinKyung Yi

**Affiliations:** 1 Department of, Microbiology and Immunology, University of Texas Medical Branch at Galveston, Galveston, Texas United States of America; 2 Department of Pathology, University of Texas Medical Branch at Galveston, Galveston, Texas United States of America; 3 Department of Internal Medicine, Division of Infectious Diseases, University of Texas Medical Branch at Galveston, Galveston, Texas United States of America; 4 Community Health, University of Texas Medical Branch at Galveston, Galveston, Texas United States of America; 5 Present Address: Department of Internal Medicine, McGovern Medical School, Houston, Texas United States of America

## Abstract

Retrospective studies indicate that co-infection of hepatitis C virus (HCV) and human immunodeficiency virus (HIV) accelerates hepatic fibrosis progression. We have developed a co-culture system (MLH) comprising primary **m**acrophages, hepatic stellate cells (HSC, **L**X-2), and hepatocytes (**H**uh-7), permissive for active replication of HCV and HIV, and assessed the effect of these viral infections on the phenotypic changes and fibrogenic gene expression in LX-2 cells. We detected distinct morphological changes in LX-2 cells within 24 hr post-infection with HCV, HIV or HCV/HIV in MLH co-cultures, with migration enhancement phenotypes. Human fibrosis microarrays conducted using LX-2 cell RNA derived from MLH co-culture conditions, with or without HCV and HIV infection, revealed novel insights regarding the roles of these viral infections on fibrogenic gene expression in LX-2 cells. We found that HIV mono-infection in MLH co-culture had no impact on fibrogenic gene expression in LX-2 cells. HCV infection of MLH co-culture resulted in upregulation (>1.9x) of five fibrogenic genes including CCL2, IL1A, IL1B, IL13RA2 and MMP1. These genes were upregulated by HCV/HIV co-infection but in a greater magnitude. **Conclusion:** Our results indicate that HIV-infected macrophages accelerate hepatic fibrosis during HCV/HIV co-infection by amplifying the expression of HCV-dependent fibrogenic genes in HSC.

## Introduction

Hepatic fibrosis is a consequence of an abnormal wound healing response to chronic liver injury, characterized by excessive production and accumulation of extracellular matrix (ECM) proteins^1^. The major cell types in the liver inducing hepatic fibrogenesis include hepatic stellate cells (HSC), hepatocytes and macrophages *(*Kupffer cells)^2–5^. Following chronic injury, quiescent HSC residing in the perisinusoidal space of Disse receive signals secreted by damaged hepatocytes, macrophages and other immune cells and undergo rapid activation into myofibroblast-like cells^5^. These cells migrate and accumulate at the sites of tissue repair, secrete pro-fibrogenic cytokines and transform into myofibroblasts expressing alpha smooth muscle actin (αSMA) and secreting large amounts of ECM proteins. This leads to disruption of equilibrium between deposition and dissolution of ECM proteins, promoting liver fibrogenesis and potentially leading to liver cirrhosis and hepatocellular carcinoma (HCC)^1,6^.

Hepatitis C virus (HCV) induces host antiviral immune responses leading to chronic inflammation that promotes hepatic fibrogenesis^7–9^. HCV-infected hepatocytes were shown to release transforming growth factor β1 (TGF-β1), one of the most potent pro-fibrotic cytokines^3^, which modulates the HSC expression of several key genes involved in liver fibrosis^10–12^. Besides the indirect effects of HCV on HSC function through infected hepatocytes, HCV proteins were shown to directly trigger HSC activation by modulating signaling and metabolic pathways^13,14^.

Due to a shared route of transmission via infected human blood, HCV and human immunodeficiency virus (HIV) co-infections are relatively common with estimated 2.3 million people living with HCV/HIV co-infection globally^15^. Several studies have demonstrated that HIV infection accelerates HCV infection-mediated hepatic fibrosis progression^16,17^. The pathogenesis of accelerated hepatic fibrosis among HIV/HCV coinfected persons is still unclear but likely complex and may include multiple factors such as direct viral effects, immune/cytokine dysregulation and augmented oxidative stress^12,18,19^.

Different *in vitro* approaches have been developed to mimic hepatic microenvironment to better understand the pathogenesis of HCV infection or HCV/HIV co-infection-mediated hepatic fibrosis. One such system was HSC monoculture incubated with heat inactivated HCV, HIV or conditioned medium from these virus infected cells^12,20^. However, monoculture systems may not recapitulate the cross talk between different hepatic cell types. Other studies employed a HSC/hepatocyte bi-culture system to study the mechanism of hepatic fibrosis caused by HCV^21^ or HIV/HCV co-infection^18^, respectively. Although these bi-culture model systems support HCV infection due to inclusion of hepatocytes, they lack macrophages (Mφ), the primary cell type supporting HIV replication. Therefore, the goal of this study was to develop a three-cell co-culture system allowing cell-cell communication between three major cell types in the liver playing central roles in hepatic fibrosis development, including HSC, hepatocytes (permissive for HCV infection) and primary Mφ (permissive for HIV infection), in order to understand the role of HCV/HIV co-infection in accelerating the hepatic fibrosis by activating HSC. Our study revealed that active replication of HIV in Mφ amplified the selective fibrogenic signals in HSC induced by HCV replication in hepatocytes under three cell co-culture condition in a Mφ-dependent manner.

## Results

### Establishment of a *novel*, co-culture model system consisting of three cell types involved in hepatic fibrosis, and supporting HCV and HIV co-infection

**Figure 1:**
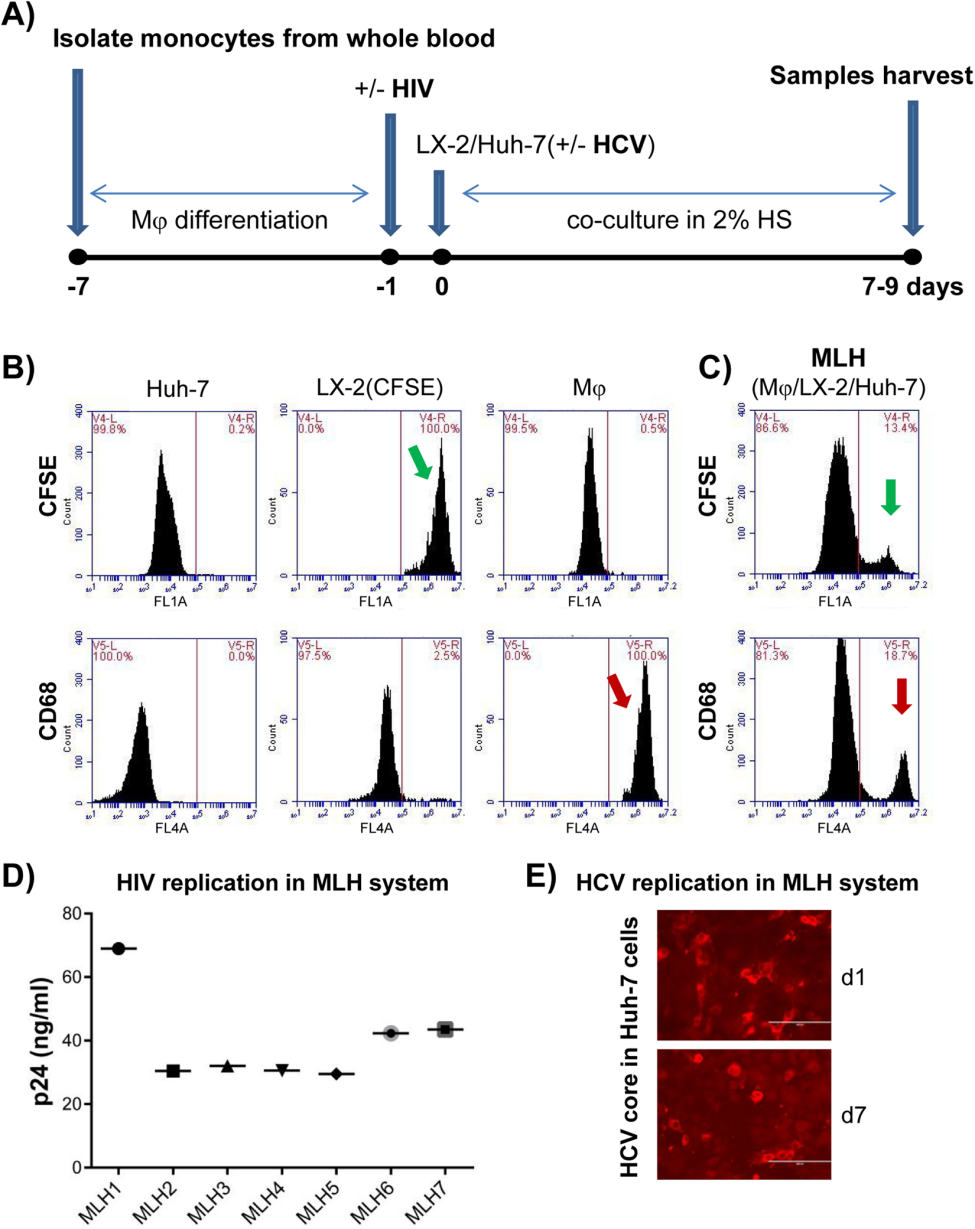
Development of three cell co-culture system (MLH) permissive for HCV and HIV replication consisting of macrophages (Mφ), hepatic stellate cells (HSC, LX-2) and hepatocytes (Huh-7). (**A**) Schematic of co-culture procedure. ‘HS’ denotes for human serum. (**B**) Huh-7, CFSE-labeled LX-2 and Alexa®647-CD68-labeled Mφ mono-cultures were subjected to FACS analysis. (**C**) FACS analysis following the MLH co-culture for 9 days. Green and red arrow indicate the detection of CFSE-labeled LX-2 and CD68-labeled Mφ at the end of co-culture. Majority of unlabeled cells belong to Huh-7 cells. (**D**) Replication of HIV under MLH co-culture for 7 to 8 days with Mφ derived from seven healthy volunteers detected by HIV p24 antigen Elisa assay. (**E**) Replication of HCV under MLH co-culture condition detected by using HCV core antigen staining.

Currently, an *in vitro* model system that represents the hepatic microenvironment permitting active HCV/HIV co-infection is not available. In an effort to determine the role of these viral replications on hepatic fibrosis progression, we have developed a three-cell co-culture system consisting of HCV-infected hepatocytes (Huh-7, human hepatocellular carcinoma derived cell line widely used in HCV research field for its high permissiveness to HCV infection^22^), HIV-infected primary macrophages (Mφ), and hepatic stellate cells [LX-2, an immortalized line of human primary HSC^23^] as schematically shown in Fig. 1A. In brief, primary human monocyte-derived Mφ were infected with HIV^24^ and then co-culture was established by addition of Huh-7 cells, with or without HCV infection, as well as LX-2 cells. These cells (**M**φ, **L**X-2 and **H**uh-7 or **MLH** co-culture) were maintained in 2% human serum in EMEM (Eagle’s Minimum Essential Medium) up to 9 days, since longer duration of cultures caused cell death. We determined the survival of all three cell types during 9 day co-culture period by performing fluorescence-activated cell sorting (FACS) analysis (Fig. 1B,C). To facilitate detection of LX-2 cells, these cells were labeled with the Carboxyfluorescein N-hydroxysuccinimidyl ester (CFSE, fluorescent cell staining dye) [(LX-2(CFSE)]. We first verified the specific detection of LX2(CFSE) and CD68-immunostained Mφ by using FACS detectors FL1 and FL4, respectively, using each of individual cell types (Fig. 1B). Then we detected the LX-2(CFSE) and CD68-immunostained Mφ as well as non-fluorescent Huh-7 cells on day 9 of co-culture by FACS analysis (Fig. 1C). These results indicate that all three cell types in MLH co-culture could survive up to 9 day of co-culture. Importantly, we detected the replication of HIV and HCV as evidenced by detection of HIV p24 and HCV core antigen for the duration of MLH co-culture (Fig. 1D,E).

**Figure 2:**
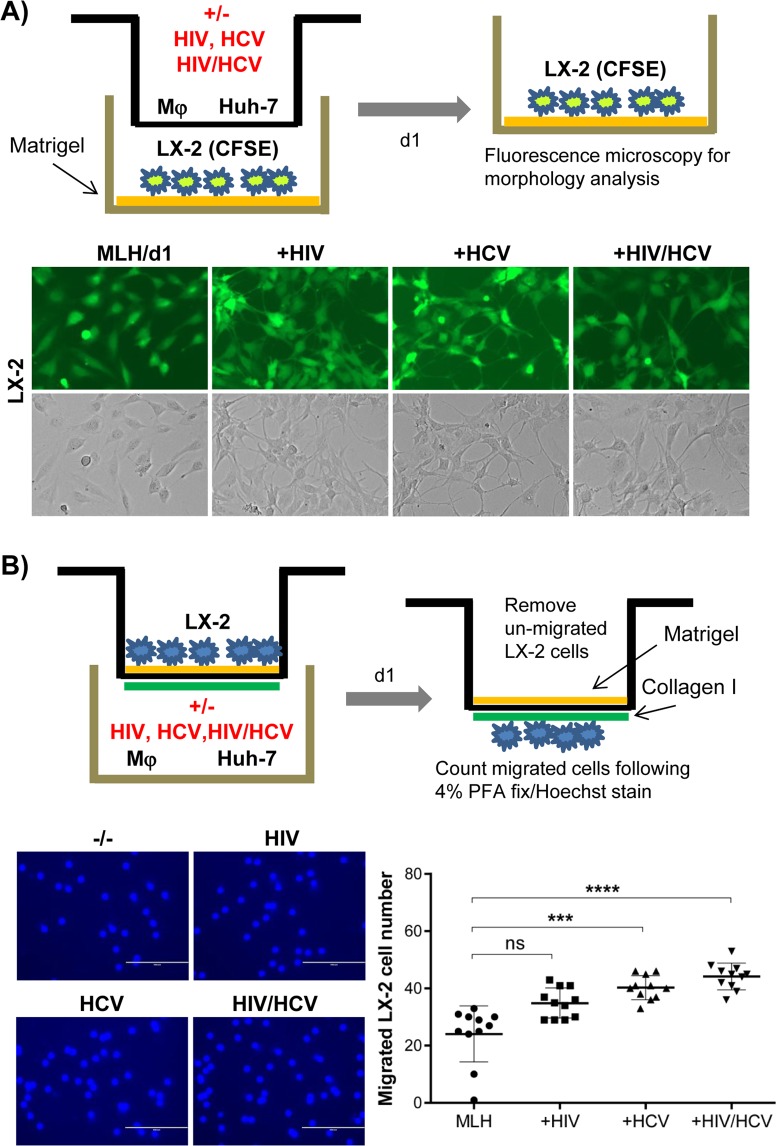
HCV or HIV replication in MLH co-culture changed the morphological and invasive phenotypes of LX-2 cells. (**A**) Schematic of trans-well system separating LX-2 cells from Huh-7 and Mφ (top). Morphological characteristics of CFSE-labeled LX2 cells following MLH co-culture with or without HIV and HCV for 24 hr (bottom). (**B**) Schematic of LX2 cell invasion assay (top). Hoechst stained LX-2 cells detected at the bottom of trans-well insert following their migration (bottom left). The numbers of migrated LX-2 cells in three independent MLH co-cultures (bottom right). Asterisks indicate statistically significant difference measured by one-way ANOVA: ****p < 0.00005; ***p < 0.0005; ns, not significant.

Activation of HSC is one of the central mechanisms of hepatic fibrosis development. Therefore, in order to determine the HSC-specific phenotypes induced by HCV and/or HIV replication, we separated LX-2 cells from Mφ and Huh-7 cells by adapting the MLH co-culture system in a transwell system as shown in Fig. 2A,B. Previous literature indicated that culturing the HSC on a plastic surface promoted their spontaneous activation, while culturing them on matrigel-coated surface allowed them to maintain their quiescence state^23,25^. Thus, we coated the LX-2 cell-culturing tissue culture plate surfaces with matrigel to maximize the detection of viral replication-dependent HSC activation phenotypes. Then we assessed the roles of HCV and or HIV infection on HSC activation phenotypes including their morphology changes, migration, proliferation and fibrogenic gene expressions^26,27^. In some cases, we used CFSE-labeled LX-2 cells to facilitate the detection of their morphology changes and proliferation.

### HIV or HCV infection promoted morphological changes in LX-2 cells and significantly enhanced their migration within 24 hr of MLH co-culture

**Figure 3:**
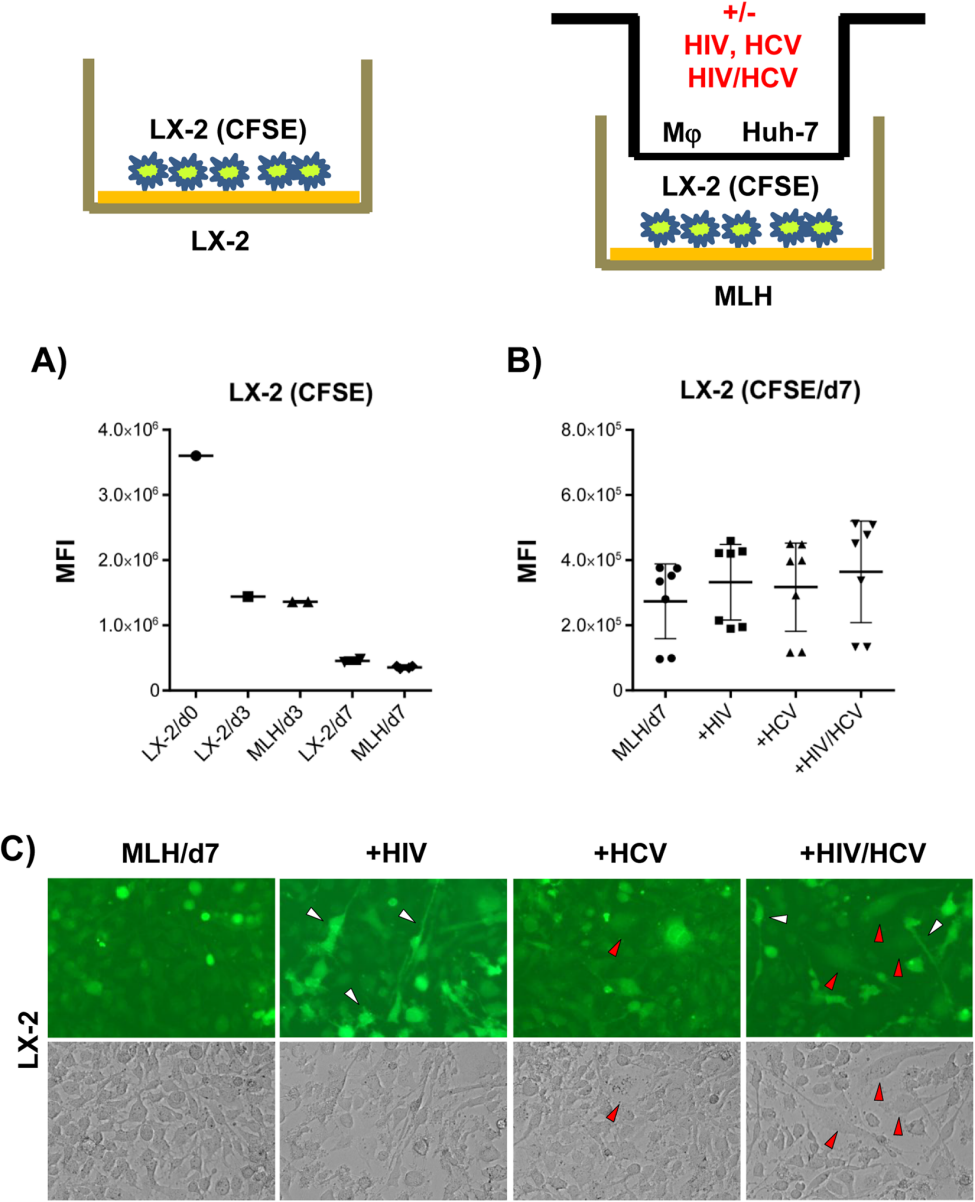
Mixed morphological changes in LX-2 cells induced by HCV/HIV co-infection of MLH co-cultures. (**A**) Proliferation of CFSE-labeled LX-2 cells during mono-culture and MLH co-culture during 7 day culture period determined by using flow cytometry. Day 0 (d0) indicates the time for initiating LX-2(CFSE) culture individually or under the condition of MLH co-culture, and the CFSE levels in LX2/d0 and MLH/d0 (data not shown) are same. **(B)** Effects of HCV and/or HIV replication in MLH co-culture on CFSE-labeled LX-2 cell proliferation determined by using FACS at day 7 of MLH co-culture. Results are from three independent experiments. **(C)** Morphology of LX-2 cells under MLH co-culture observed at day 7 by using a fluorescence and phase-contrast microscope. **(C)** LX-2 cell morphology under MLH co-culture at day 7 detected using a fluorescent and phase-contrast microscope.

We determined the effect of HCV and/or HIV replication under transwell MLH co-culture condition on LX-2 cell morphology by using a fluorescence microscope as schematically shown in Fig. 2A top panel, in which LX-2 cells were placed at the matrigel-coated bottom well while Mφ and Huh-7 cells were placed in the transwell insert. Results showed that mono- or co-infection of HCV and HIV in MLH co-culture induced distinct morphological change in LX-2 cells resulting in “stellate morphology” with elongated cytoplasmic processes, on day 1 of MLH-co-culture, compared to those lacking virus exposure, which showed flat morphology (Fig. 2A, bottom panel). The stellate morphology of LX-2 cells likely indicate their quiescent state as shown in previous report^28^, since we detected reduced proliferation of LX-2 cells showing stellate morphology (see below, Fig. 3C). Ikeda *et al*. showed that quiescent HSC migration was associated with their stellate morphology^29^. Therefore, we determined the effect of HIV and HCV replication on LX-2 cell migration efficiency. LX-2 cell migration was determined in a transwell configuration mimicking normal liver environment for HSC migration, in which the top of porous transwell membrane were coated with matrigel and bottom with collagen I to mimic space of Disse and fibrillary matrix, respectively, as described by Hu *et al*.^30^ (Fig. 2B, top panel). We placed LX-2 cells on top of matrigel coated transwell insert and counted the number of LX-2 cells migrated to the bottom of transwell within a day of their co-culture with Mφ and Huh-7 cells placed at the bottom well, in the presence and absence of HCV and/or HIV infection (Fig. 2B, bottom left panel). The data generated by using three different MLH co-cultures, derived by using three independent primary Mφ preparations, showed that HCV mono-infection and HCV/HIV co-infection, not HIV infection, significantly enhanced LX-2 cell migration compared to no viral infection (Fig. 2B, bottom right panel).

### HCV/HIV co-infection of MLH culture for 7 days did not affect overall LX-2 cell proliferation

We have measured the rate of LX2(CFSE) cell proliferation, by determining the fluorescence intensity of CFSE levels, to be ~three divisions during 7-day culture period, regardless of whether LX-2 cells were cultured singly or under MLH co-culture condition in a transwell setting (Fig. 3A).

Next, we determined the effect of HIV or HCV infection, or HCV/HIV co-infection, on LX2(CFSE) cell proliferation and morphology change under MLH co-culture/transwell setting following 7-day co-culture. As shown in Fig. 3B, overall LX-2 cell proliferation was not affected by HCV and HIV mono- or co-infection of MLH co-culture. However, fraction of LX-2 cells showing stellate morphology in HIV and or HCV/HIV co-infected MLH co-cultures retained higher levels of CFSE compared to cells showing a “round” morphology, indicating a reduced rate of proliferation (Fig. 3C, white arrowheads). Also, LX-2 cells from HCV/HIV co-infected MLH co-cultures showed a higher incidence of an elongated and flattened myofibroblast-like morphology (Fig. 3C, red arrowheads). This myofibroblast-like morphology in LX-2 cells was also detectable from HCV-infected MLH co-cultures, although less frequently. The mixed presence of the LX-2 cell population, showing both quiescence-associated stellate morphology and proliferation-associated myofibroblast-like morphology in HCV/HIV co-infected MLH co-culture (Fig. 3C, see also the review by Anthony *et al*.^28^), likely resulted in the lack of significant net effect of HCV/HIV infection on LX-2 cell proliferation (Fig. 3B). In summary, these results suggest that co-infection of HCV/HIV induced distinct, mixed morphological and proliferative phenotypic changes in LX-2 cells under MLH co-culture condition.

### HIV/HCV co-infection augmented the HCV infection-dependent upregulation of selected fibrogenic genes in LX-2 cells under MLH co-culture conditions

**Figure 4:**
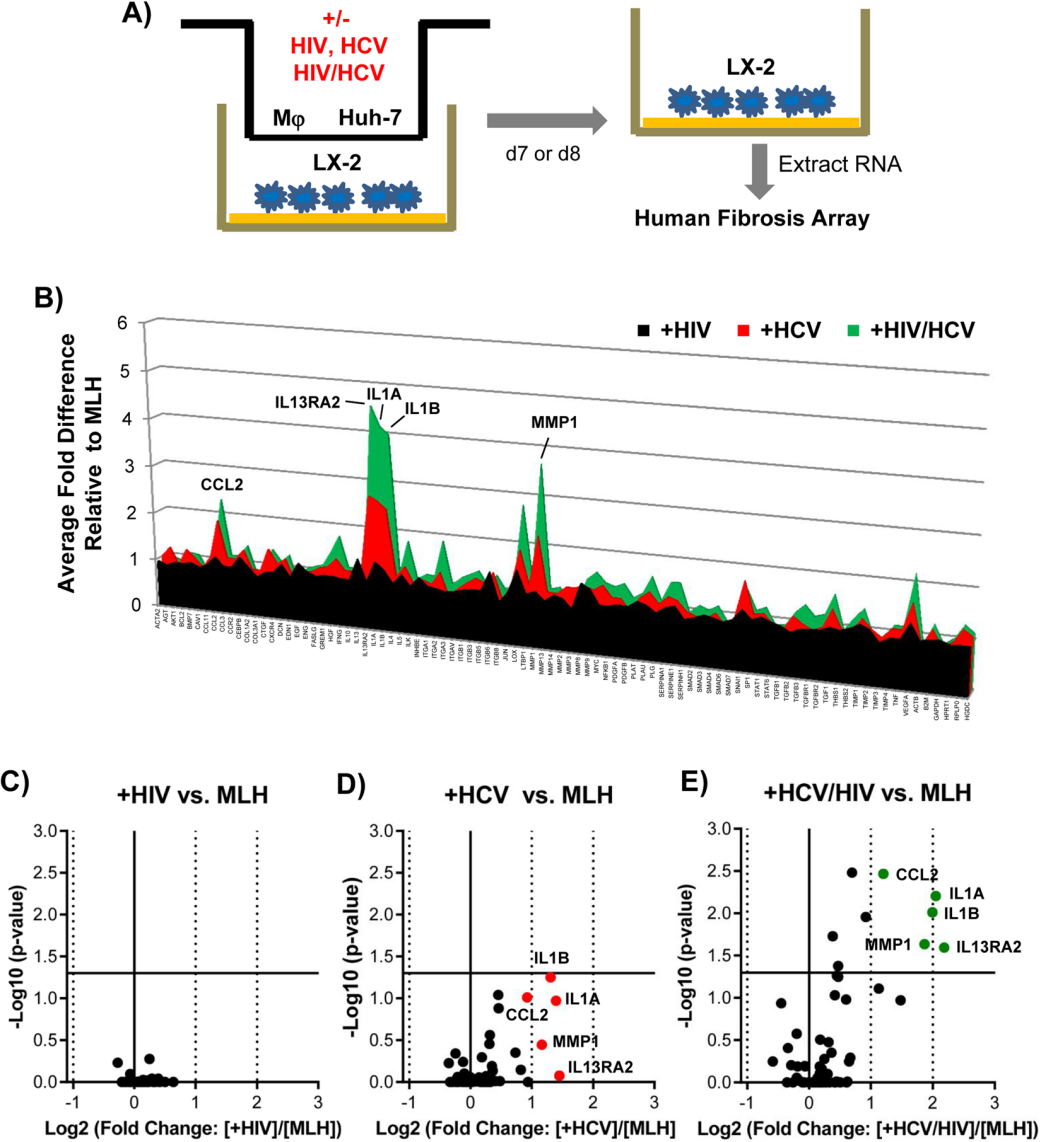
HCV/HIV co-infection augmented the induction of specific fibrogenic genes upregulated by HCV in MLH co-culture system. (**A**) Schematic of human fibrosis micro array by using LX-2 cell RNA isolated following MLH-co-culture with or without HCV and HIV. **(B)** Relative gene expression fold changes in LX-2 cells detected in the microarray analysis between non-infected and HIV-, HCV- and HCV/HIV-infected MLH co-cultures, respectively. A volcano plots showing the deregulated genes in LX-2 cells following **(C)** HIV, **(D)** HCV or **(E)** HCV/HIV infection of MLH co-culture. Genes up-regulated more than 1.9 folds by HCV and HCV/HIV infection are shown in red and green dots, respectively.

**Figure 5:**
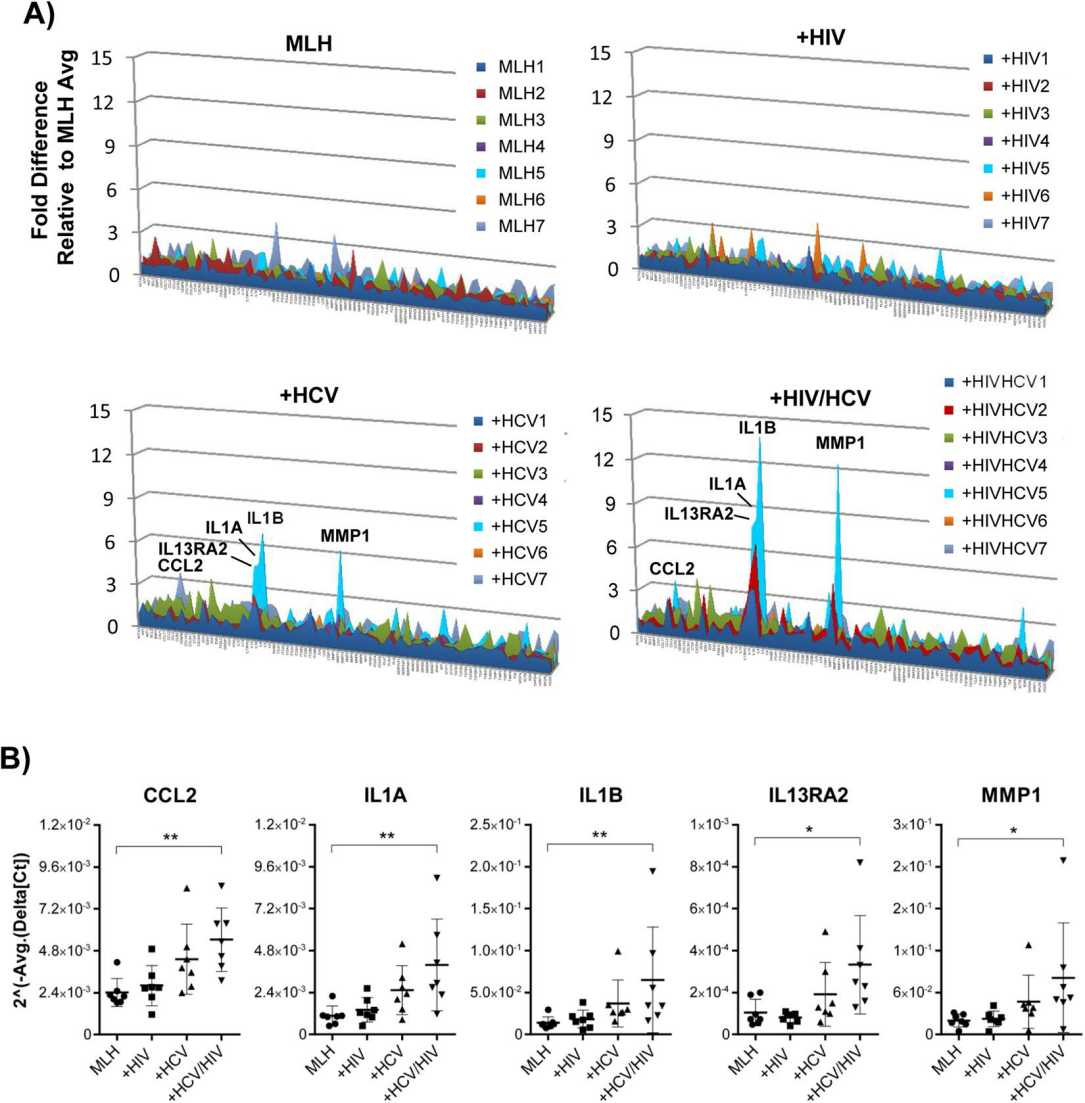
Primary Mφ in MLH co-cultures regulated the magnitude of HCV- or HCV/HIV-dependent fibrogenic gene induction in LX-2 cells. (**A**) Gene transcript levels in LX-2 cells from MLH co-cultures uninfected or infected with HCV and HIV, normalized to average values of individual genes from uninfected MLH co-cultures. (**B**) Individual values of normalized gene expression levels of five fibrogenic genes whose expression were upregulated more than 1.9 fold on average by HCV infection following 7 independent MLH co-cultures. Asterisks indicate statistically significant difference measured by one-way ANOVA: **p < 0.005; *p < 0.05.

To assess the effect of HCV and HIV mono- or co-infection on fibrogenic gene expression in LX-2 cells, we isolated total RNA from LX-2 cells cultured in transwell MLH co-culture conditions in the presence and absence of HCV and/or HIV infection, and then subjected these RNAs to human fibrosis microarray analysis (Fig. 4A). Initial assessment indicated that the effects of viral infection in MLH co-culture on fibrogenic gene expressions in LX-2 cells became evident by day 7 post-co-culture (Supplementary Fig. S1A). Therefore, we performed all subsequent microarray experiments by using LX-2 cell RNAs collected on day 7 to 8 of MLH co-cultures (generated by using 7 different primary Mφ) in the presence or absence of HCV and or HIV infection. The results of these experiments were following: After HCV mono-infection, five genes, including C-C motif chemokine ligand 2 (CCL2), interleukin 13 receptor subunit alpha 2 (IL13RA2), interleukin 1α (IL1A), interleukin 1β (IL1B) and matrix metalloprotease 1 (MMP1) were upregulated, on average, more than 1.9 fold in LX-2 cells during MLH co-culture. (Fig. 4B, red fill). However, their upregulations were not statistically significant as shown in volcano plot (Fig. 4D, red dots). Interestingly, these same genes were also upregulated by HCV/HIV co-infection but in greater magnitude (Fig. 4B, green fill) and statistically significant manner (Fig. 4E, green dots). On the contrary, HIV mono-infection showed no impact on fibrogenic gene expression in LX-2 cells under equivalent conditions (Fig. 4B, black fill. See also Fig. 4C). Clustergram shown in Supplementary Fig. S1B summarizes the relatedness between HCV infection- and HCV/HIV co-infection-mediated changes in fibrogenic gene expression patterns in LX-2 cells from MLH co-culture, compared to no infection, as well as lack of obvious changes by HIV infection. In general, primary Mφ from different donors had minimal to moderate variation in most fibrogenic gene expression levels in LX-2 cells during MLH co-cultures regardless of viral infection (Fig. 5A, see also Supplementary Fig. S2). However, profound variations in the expression levels of above-mentioned, five selective genes in LX-2 cells were detected following infection of HCV or HCV/HIV to different MLH co-cultures differing only by their primary Mφ content (Fig. 5A,B).

These results suggest that HCV infection is the main driver of selective fibrogenic gene upregulation in LX-2 cells under MLH co-culture condition and HIV-co-infection augmented this HCV-mediated gene upregulation. Importantly, these results suggest that variation in primary Mφ, which was the only variable within MLH co-culture constituents, determined the extent of selective fibrogenic gene upregulation in LX-2 cells under MLH co-culture infected by HCV or HCV/HIV.

### HCV or HCV/HIV co-infection did not affect alpha smooth muscle actin (αSMA) gene transcription and protein expression

**Figure 6:**
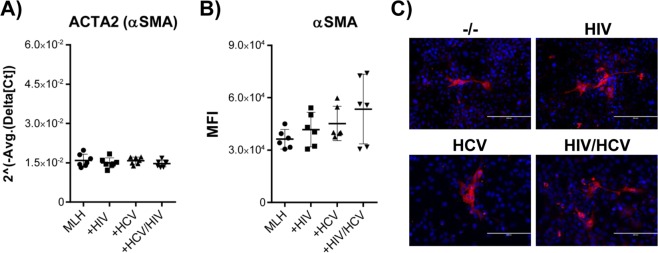
Effect of HCV and/or HIV infection in MLH on αSMA expression in LX2 cells. (**A**) Normalized gene expression levels of αSMA gene in LX2 cells from seven different MLH co-cultures in the presence or absence of HCV and HIV. **(B)** FACS analysis of αSMA protein expression profile in LX-2 cells on day 7 of three independent MLH co-cultures with or without HCV and HIV infection. **(C)** Immunofluorescence analysis of αSMA in LX2 cells on day 7 of MLH co-cultures in the presence or absence of HCV and HIV.

Induction of αSMA has been considered as one of the most reliable markers of stellate cell activation^27^. However, infection of MLH co-culture by HCV or HIV had altered neither ACTA2 (Smooth muscle aortic alpha-actin) gene transcript levels nor αSMA proteins expression levels in LX-2 cells, determined by quantitative RT-PCR (Fig. 6A) and FACS analysis (Fig. 6B), respectively. In addition, immunofluorescence analyses indicate that only small proportion of LX-2 cells expressed detectable level of αSMA protein under our MLH co-culture condition on day 7 (Fig. 6C).

## Discussion

In this study, we have developed an *in vitro* co-culture system (MLH) consisting of three major cell types in the liver involved in hepatic fibrosis development, including primary Mφ, HSC (LX-1) and hepatocytes (Huh-7), permissive for active replication of HCV and HIV. We have determined the effects of HCV and HIV on early phenotypic changes in HSC to understand the viral mechanisms triggering HSC activation and resulting in hepatic fibrosis. Our results indicated that HCV and/or HIV replication in MLH co-cultures trigger morphological changes in HSC and enhanced the invasive potential of HSC (Fig. 2). Importantly, HIV mono infection in MLH co-culture had no impact on fibrogenic gene transcriptions in HSC, while co-infection of HCV/HIV significantly augmented the expression of HCV-responsive fibrogenic genes in HSC, including CCL2, IL1A, IL1B, IL13RA2 and MMP1 (Figs 4 and 5), providing mechanistic insight into enhanced fibrogenesis in HCV/HIV co-infected patient population compared to those infected with HCV alone.

Use of different primary Mφ in our MLH co-culture system provided us with a perspective regarding the role of different Mφ for upregulation of fibrogenic genes in HSC upon HCV and/or HIV infection. Interestingly, expression of the majority of fibrogenic genes in HSC showed limited variations between MLH cultures generated by using Mφ from different donors, regardless of HCV and/or HIV infection under our experimental condition (Figs 5A and S2). However, in the case of five selective genes upregulated 1.9 fold or more on average by HCV or HCV/HIV co-infection, the magnitude of these gene inductions in HSC varied substantially depending on different primary Mφ used (Fig. 5A,B). It is likely that differences in individual phenotypes of Mφ contributed to this variation, since previous studies suggested that HCV-dependent induction of M2-polarized Mφ promoted HSC activation and hepatic fibrosis^25,31,32^. Interestingly, we detected no correlation between HIV replication levels (represented by p24 levels in Fig. 1D) and altered fibrogenic gene expression folds in LX-2 cells following HIV infection of different MLH co-culture (Fig. 5A). These results suggest that divergent primary Mφ phenotypes following HIV infection, not the HIV replication efficiency *per se*, determined the magnitude of HCV/HIV co-infection-dependent fibrogenic gene expression in LX-2 cells under MLH co-culture condition. Future studies are needed to determine the Mφ phenotypes that will trigger high level of fibrogenic gene expression in HSC following HCV or HCV/HIV co-infection. Such information could be useful to identify patients at higher risk of developing hepatic fibrosis following these viral infections.

Theoretically, HIV-infected Mφ could have directly modulated fibrogenic gene expression in HSC regardless of HCV infection or induced a different set of genes from those induced by HCV in the coinfection condition and thereby accelerate hepatic fibrosis in HCV/HIV co-infection cases. However, by performing microarray analysis of 84 genes involved in human fibrosis using HSC specific RNA samples obtained from MLH co-culture system, our study eliminated either of these possibilities. Instead, our data showed that HCV/HIV co-infection augmented fibrogenic gene expression in HSC compared to HCV mono infection, consistent with previous report^18^. However, different from this previous study^18^, we did not detect the effect of HIV on fibrogenic gene expression in HSC. Differences in experimental strategies such as HSC/hepatocytes exposed to HIV in the previous study versus MLH that include primary Mφ infected with HIV in our study, may have caused this discrepancy.

Under our experimental conditions, well-known fibrogenic genes, including αSMA (ACTA2), isoforms of collagenase (COL1A2 and COL3A1), transforming growth factor-beta (TGF-β), tissue inhibitor of metalloproteinase (TIMP, except TIMP1), and matrix metalloproteinase (MMP, except MMP1) were not induced in LX-2 cells by HCV and HIV mono- and co-infection of MLH co-cultures (Figs 6 and S2). Despite this, we detected myofibroblast-like morphological changes in fraction of HSC from HCV infected or HCV/HIV co-infected MLH co-culture at 7 days post co-culture indicating that those HSC may have undertaken an activation process (Fig. 3C).

It is notable that substantially upregulated fibrogenic genes in HSC by HCV or HCV/HIV infection of MLH co-culture belong to inflammatory cytokines, including CCL2, IL1A, IL1B and IL13RA2. The CCL2, also called monocyte chemoattractant protein 1 (MCP1), is a proinflammatory cytokine secreted by HSC and Kupffer cells in the liver, and promotes hepatic fibrosis by stimulating the recruitment of monocytes to the injured liver^33,34^. Based on our data, we propose that significant upregulation of CCL2 in HSC under HCV/HIV co-infection condition likely contributed to enhanced fibrosis development, in addition to previously suggested immuno-pathological mechanism of CCL2 for accelerating HCV/HIV co-infection-mediated liver fibrosis^35^.

IL1A and IL1B constitute two forms of the proinflammatory cytokine interleukin-1. Previous studies suggested IL1A as an early responder of inflammatory response and IL1B as a late responder, recruiting neutrophils and Mφ, respectively, to the site of injury^36^. IL1 receptor knockout mice were protected from hepatic fibrosis development indicating the critical roles of these two cytokines in this process^37^. Our data indicate that significant upregulation of IL1A and IL1B from quiescent HSC due to HCV/HIV co-infection might be an early inflammatory response contributing to enhanced HSC activation leading to hepatic fibrosis.

IL13RA2 was shown to be overexpressed in activated HSC, and blocking the IL13 receptor reduced hepatic fibrosis development caused by non-alcoholic steatohepatitis (NASH)^38^. Our data indicate that HCV infection could promote IL13RA2 expression in HSC, which could be further upregulated by HIV co-infection, supporting the role of this factor in accelerated hepatic fibrosis by HCV/HIV co-infection.

Matrix metalloproteinases (MMPs) degrade extracellular matrix and play critical roles in tissue repair and remodeling. Different MMPs were implicated to function differently based on their substrate specificity, and environment in which they are expressed, not only in ECM remodeling but also in immune responses^39^. Over expression of MMP-1, MMP-8 and MMP-13 was shown to reduce the number of activated HSC and attenuate the hepatic fibrosis when transiently over expressed in the liver^40–43^. These findings suggest the protective role of MMPs during liver injury. However, evidence for profibrotic roles of MMPs also exist^44^. Since HCV-dependent MMP-1 induction in HSC was augmented by HIV co-infection, we speculate that MMP1 induction in HSC could have a potential to play a profibrotic role.

HCV-infected hepatocytes and HIV-infected Mφ were separated from HSC via transwell during our three cell co-culture condition. Therefore, soluble factors from HCV and HIV infected hepatocytes and Mφ must have contributed to the induction of fibrogenic genes in HSC. One such candidate is TGF-β1, which plays a pivotal role during tissue fibrosis development^45^, since HCV replication in Huh-7 cells was shown to induce this cytokine^46–48^. However, the study by Schulze-Krebs indicates that TGF-β1accounted for only ~50% of profibrogenic activity derived from HCV replicating cells, suggesting the presence of additional mediators induced by HCV replication^48^. Interestingly, recent literature idicates the important role of TGF-β2 in hepatic stellate cells activation and liver fibrogenesis, potentially even more so than the role of TGF-β1 in this process^49–51^. Chida *et al*. showed that silencing of TGF-β2 in HCV-infected Huh-7 cells reduced fibrogenic phenotypes in the human hepatic stellate cell line TWNT4 in two cell co-culture study. Importantly, they showed that serum TGF- β2 levels in HCV-infected patients positively correlated with hepatic fibrosis stages F0-F2. Supporting this finding, a recent study by Abd el-Meguid *et al*. also demonstrated positive correlations between HCV infection-associated hepatic fibrosis and elevated TGF-β2 level in serum and peripheral leucocytes^51^. Our preliminary data showed the significantly higher TGF-β2 levels in supernatant of MLH co-culture following HCV/HIV-co-infection compared to no infection (Supplementary Fig. S3). These results suggest that TGF-β2 may be one of the main drivers of HCV/HIV-co-infection mediated upregulation of fibrogenic genes in HSC. However, further studies are necessary to establish the exact role of TGF-β2 in HCV/HIV co-infection mediated acceleration of hepatic fibrosis development. Also effort should be directed to identify any additional, soluble profibrogenic factors responsible for activating HSC during HCV/HIV co-infection, since such biomarkers could serve as a diagnostic tool to detect hepatic fibrosis induction or a potential target of therapeutic interventions to inhibit fibrosis development following these viral infections.

One of the limitations of this study is that our *in vitro* system does not include other residential liver components, such as sinusoidal endothelial cells, which were shown to contribute to hepatic fibrosis development^52^. Additional limitations may be the small number of primary Mφ that we used to generate MLH co-cultures, which limited the representation of Mφ characteristics from free-range, out bred human population. Despite these limitations, the three-cell MLH co-culture system, for the first time, allowed us to determine the effects of active replication of HCV and HIV in hepatocytes and Mφ, respectively, on fibrogenic gene expression in hepatic stellate cells associated with hepatic fibrosis development.

In conclusion, we showed that HCV infection in hepatocytes trigger key fibrogenic factors in HSC, including proinflammatory cytokines as well as factors involved in tissue remodeling leading to activation of HSC. HIV infection in Mφ, while having no impact on fibrogenic gene expression in HSC, specifically augmented HCV-dependent fibrogenic factor induction in HSC. We believe that our data provided mechanistic insight into accelerated hepatic fibrosis by HCV/HIV co-infection, which is HIV-dependent amplification of HCV-mediated HSC activation.

## Methods

### Cell lines and culture conditions

The Huh-7 cell line was derived from human hepatocellular carcinoma and expressed α-fetoprotein (AFP)^53^ without any evidence of viral infection. This cell line has been widely used in HCV reserch field for its high permissiveness to HCV infection^22^. Huh-7 cells were maintained in DMEM with 10% FBS at 37 °C in 5% CO_2_ conditions. LX-2 cells are immortalized primary human hepatic stellate cell line, and were purchased from EMD Millipore and maintained in DMEM with 2% FBS at 37 °C in 5% CO_2_ conditions according to the manufacturer’s recommendation.

### Monocytes culture

Peripheral blood mononuclear cells (PBMCs) were isolated from healthy human donors under the UTMB Institutional Review Board (IRB)-approved protocol, by density gradient centrifugation over Ficoll. Informed consent was obtained from all participants. Purified monocytes were resuspended and cultured in medium consisting of Iscove’s Modified Dulbecco’s Media **(**IMDM) supplemented with 2% human AB serum (Invitrogen, Madison, WI), 1% Penn-Strep at 37 °C in 5% CO_2_ conditions, then differentiated to macrophages by incubating them for 7 days in complete medium supplemented with 50 ng/ml GM-CSF.

### Transwell co-culture

For co-culture experiments, primary macrophages (+/−HIV) and Huh-7 cells (+/−HCV) were placed in hanging cell inserts of 6 well transwell (Corning Incorporated, Acton, MA, 0.4 µm pore size) at a seeding density of 1 × 10^5^/well and 2.5 × 10^5^/well, respectively. LX2 cells were labeled with CFSE (Carboxyfluorescein diacetate succininyl ester). Briefly, LX2 cells were incubated with CFSE (5uM) for 20 min at 37 °C, then washed twice with PBS, once with DMEM (10% FBS) and finely incubated with DMEM (2% FBS). CFSE-labeled LX-2 cells were seeded on top of matrigel layer in bottom well at a seeding density 5 × 10^4^/well. The co-culture media conditions were optimized based on our strategy to ensure the balanced survival of all three cell types during co-culture period lasting 7 to 9 days. We chose to use primary macrophage culture medium (IMDM, 2% human serum and 1% Penn-Strep) to reduce the relative over growth of LX-2 cells and Huh-7 cells in comparison to non-dividing primary macrophages. Original seeding numbers of different cell types were determined based upon the growth rates of Huh-7 and LX-2 cells to give the most biologically relevant ratio of cell numbers possible throughout the duration of co-culture under *in vitro* experimental condition^54,55^.

### Transwell invasion assay

LX2 were seeded in the hanging insert of transwell plate (0.8 µm pore size), which were pre-coated with collagen (100 µg/ml) and matrigel (50 µg/ml). Macrophages (+/−HIV) and Huh-7 cells (+/−HCV) were seeded in the lower compartment. After incubating the assembled transwell plates at 37 °C for 24 hrs, migrated LX2 cells were counted by staining cell nuclei with Hoechst.

### HIV-1 infection of macrophages

Mφ were infected with HIV (SF162) at an MOI of 0.02 for 24 hrs and then washed and cultured in RPMI1640 containing 2% human AB serum and 1% Penn-Strep at 37 °C and 5% CO_2_.

### HIV p24 capture ELISA

HIV p24 level was measured by ELISA using plates coated with a monoclonal antibody to HIV-1 p24 for capturing HIV-1 p24 antigen [ImmunoDX, LLC (IDX)] according to the suggested protocol. Absorbance was recorded at 450 nm.

### HCV RNA Electroporation

Genotype 1a H77S RNA was electroporated into Huh-7 cells as previously described^56^. HCV infection was determined by detecting HCV core using indirect immunofluorescence.

### Gene expression analysis by RT^2^ PCR profiler Array

The RT^2^ PCR Profiler (SA Biosciences, Qiagen) was used to examine the expression patterns of 84 genes involved in human fibrosis, according to the manufacturer’s instructions by using RNA isolated from LX-2 following MLH co-cuture with or without HCV and HIV. The Real-time RT-PCR was performed in a Bio-Rad PCR machine (model CFX96). Gene expression fold difference was analyzed for those genes whose Ct value was less than 34 by using the web-based software RT^2^ Profiler PCR Array Data Analysis (Qiagen) (see Supplementary data set file for detail).

### Statistical Analysis

The one-way analysis of variance (ANOVA) was performed by using GraphPad Prism 6 software to determine the significance in differences between non-infected and HCV and/or HIV infected samples. In brief, we first performed the Shapiro-Wilk test to determine whether the data show the normal distribution. For the data showing the normal distribution, we have performed ordinary one-way ANOVA. For those failed to pass the normal distribution test, we have performed Kruskal-Wallis test (see Supplementary data set file for detail).

### Immunofluorescence staining

Cells grown on matrigel coated 24-well plates/8 well chambers were fixed with 4% paraformaldehyde, permeabilized and then incubated with primary antibodies [anti HCV core (MA1-080, Invitrogen, Rockford) or anti αSMA (61001, PROGEN Biotechnik, Heidelberg)] in permeabilization solution (0.1% Triton in PBS supplemented with 3% BSA) for 1 hr, and after three washes, incubated with fluorescently-labeled secondary antibodies (anti-mouse Alexa Fluor 647, Invitrogen, Rockford) and Hoechst 33258 (83219, Anaspec Inc, Fremont) for 1 hr. Samples were observed either with Fluoview FV10i confocal microscope (Olympus) or Evos immunofluorescence microscope (life technologies).

### Flow Cytometry

CFSE pre-labeled LX2 cells were stained with αSMA in PBS supplemented with 3% BSA and 0.1% Triton X-100 for 1 hr following fixation  with 4% paraformaldehyde and subjected to fluorescence-activated cell sorter (FACS) analysis by using an Accuri™ C6 Cytometer (BD Biosciences).

## Supplementary information

Supplementary Information

Dataset 1

## Data Availability

The datasets generated and/or analyzed during the current study are available from the corresponding author on reasonable request.
