# Supplementary material for: Fibrogenic Gene Expression in Hepatic Stellate Cells Induced by HCV and HIV Replication in a Three Cell Co-Culture Model System
Source: Sci Rep. 2019 Jan 24;9:568. doi: 10.1038/s41598-018-37071-y (PMC6345841; doi:10.1038/s41598-018-37071-y)
Supplement: Supplementary file 1 — Supplementary Information [file 41598_2018_37071_MOESM1_ESM.docx]

**SUPPLEMENTARY INFORMATION**

**Fibrogenic Gene Expression in Hepatic Stellate Cells Induced by HCV and HIV Replication in a Three Cell Co-Culture Model System**

Abdellah Akil^1^, Mark Endsley^1^, Saravanabalaji Shanmugam^1^, Omar Saldarriaga^2^, Anoma Somasunderam^3,#^, Heidi Spratt^4^, Heather L. Stevenson^2^, Netanya S. Utay^3,#^, Monique Ferguson^3^ and MinKyung Yi^1,*^

^1^Department of ^1^Microbiology and Immunology, ^2^Department of Pathology, ^3^Department of Internal Medicine, Division of Infectious Diseases and ^4^Community Health, University of Texas Medical Branch at Galveston, Galveston, Texas, United States of America.

# Current address: Department of Internal Medicine, McGovern Medical School, Houston, Texas, United States of America.

*Corresponding, [miyi@utmb.edu](mailto:miyi@utmb.edu)


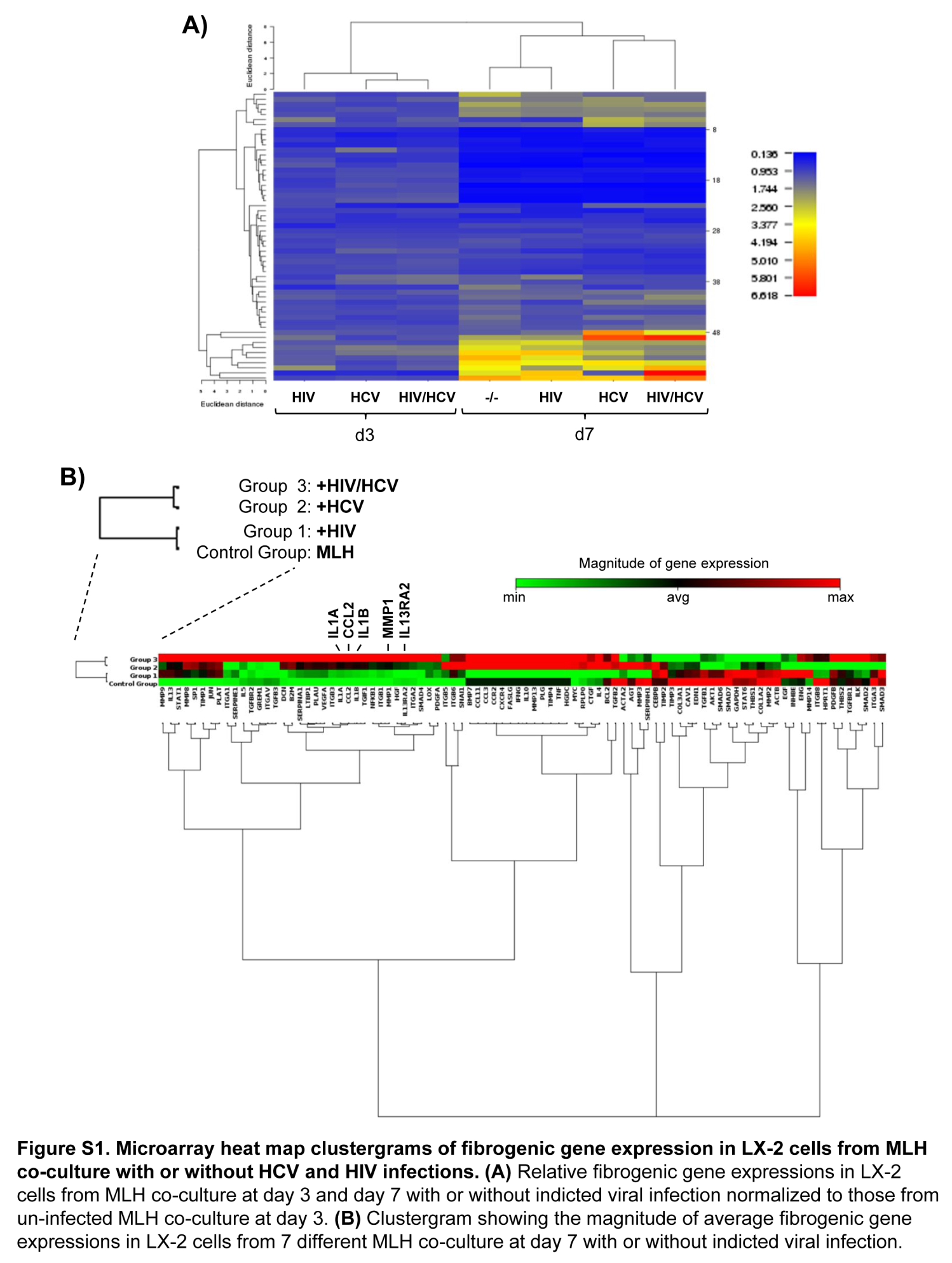


**Supplementary Figure S1. Microarray heat map clustergrams of fibrogenic gene expression in LX-2 cells from MLH co-culture with or without HCV and HIV infections. (A)** Relative fibrogenic gene expressions in LX-2 cells from MLH co-culture at day 3 and day 7 with or without indicted viral infection normalized to those from un-infected MLH co-culture at day 3. **(B)** Clustergram showing the magnitude of average fibrogenic gene expressions in LX-2 cells from 7 different MLH co-culture at day 7 with or without indicted viral infection.


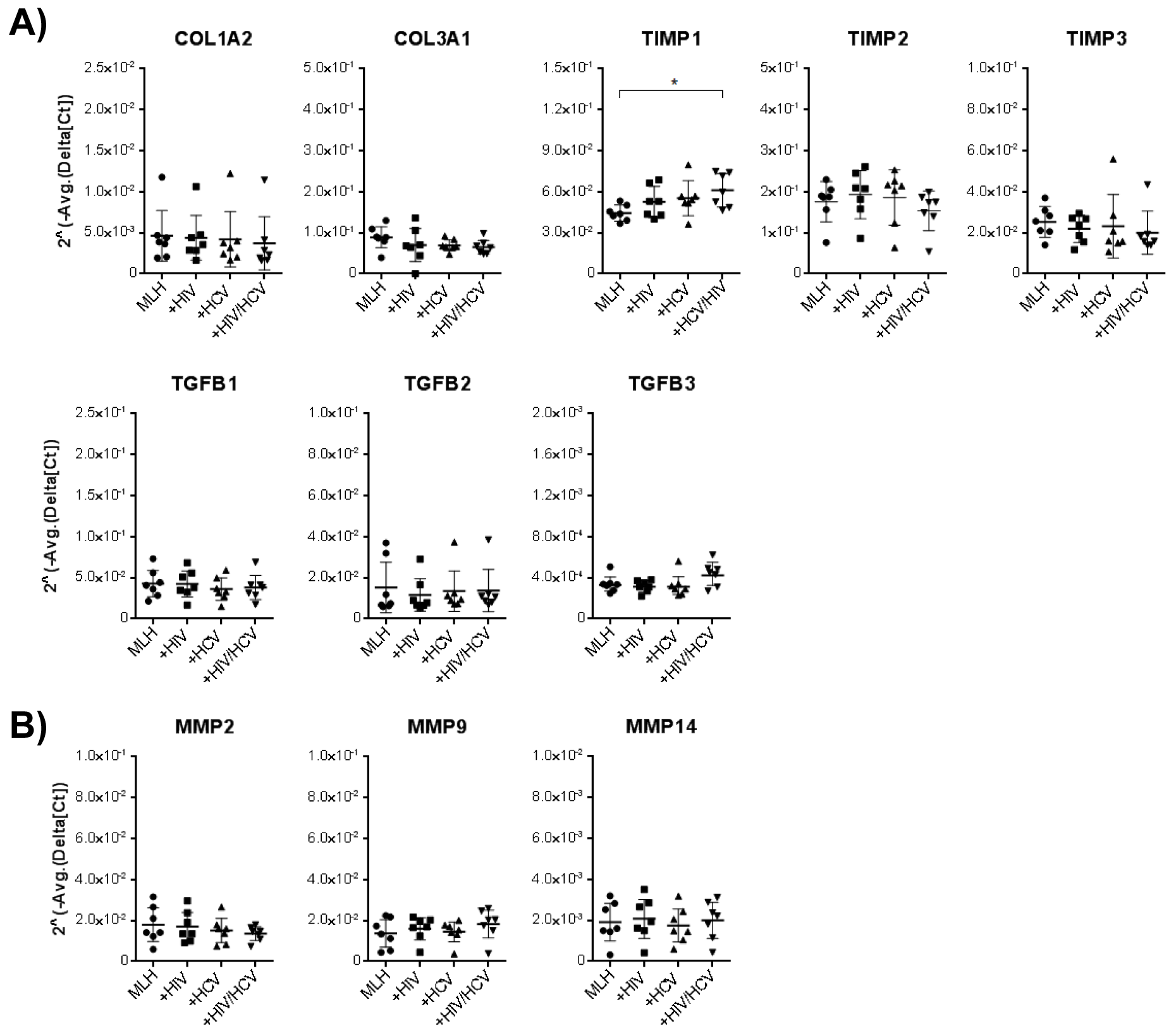


**Supplementary Figure S2. Average gene transcript levels of COL1A2, COL3A1, TIMP1, TIMP2, TIMP3, TGFB1, TGFB2, TGFB3, MMP2, MMP9, MMP14 derived from microarray analysis of LX2 cell RNA from 7 independent MLH co-culture system in the presence or absence of HCV and HIV infection.** Asterisk indicates statistically significant difference between values from uninfected and infected with viruses in one-way ANOVA: *, p<0.05.

###
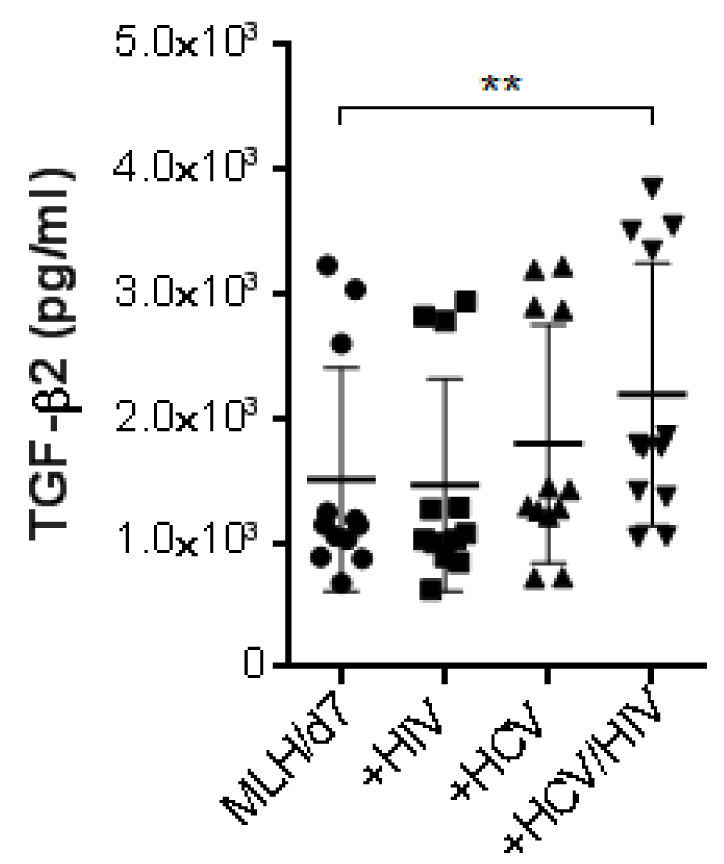


### Supplementary Figure S3. TGF-β2 protein levels in the supernatant of MLH co-cultures infected with or without HCV and HIV. The TGF-β2 level in the supernatant from MLH co-cultures with or without HCV and HIV infection collected on day 7 of co-culture were measured by using ELISA. The results are from three independent MLH co-culture studies. Asterisk indicates statistically significant difference between values from uninfected and infected with viruses by Friedman test in one-way ANOVA: **, p<0.005.
